# Supplementary material for: Characterization of p53 p.T253I as a pathogenic mutation underlying Li-Fraumeni Syndrome
Source: PLoS One. 2025 Dec 5;20(12):e0320036. doi: 10.1371/journal.pone.0320036 (PMC12680149; doi:10.1371/journal.pone.0320036)
Supplement: S1 Table — (PDF) [file pone.0320036.s001.pdf]

S1 Table

Antibody	Manufacturer	Cat. #
p53	Cell Signaling Technologies	2527S
p21	Cell Signaling Technologies	2947S
MDM2	Cell Signaling Technologies	86934S
GAPDH	Cell Signaling Technologies	2118L
H3	Cell Signaling Technologies	D2B12
