# Supplementary figures and images for: Characterization of p53 p.T253I as a pathogenic mutation underlying Li-Fraumeni Syndrome
Source: PLoS One. 2025 Dec 5;20(12):e0320036. doi: 10.1371/journal.pone.0320036 (PMC12680149; doi:10.1371/journal.pone.0320036)

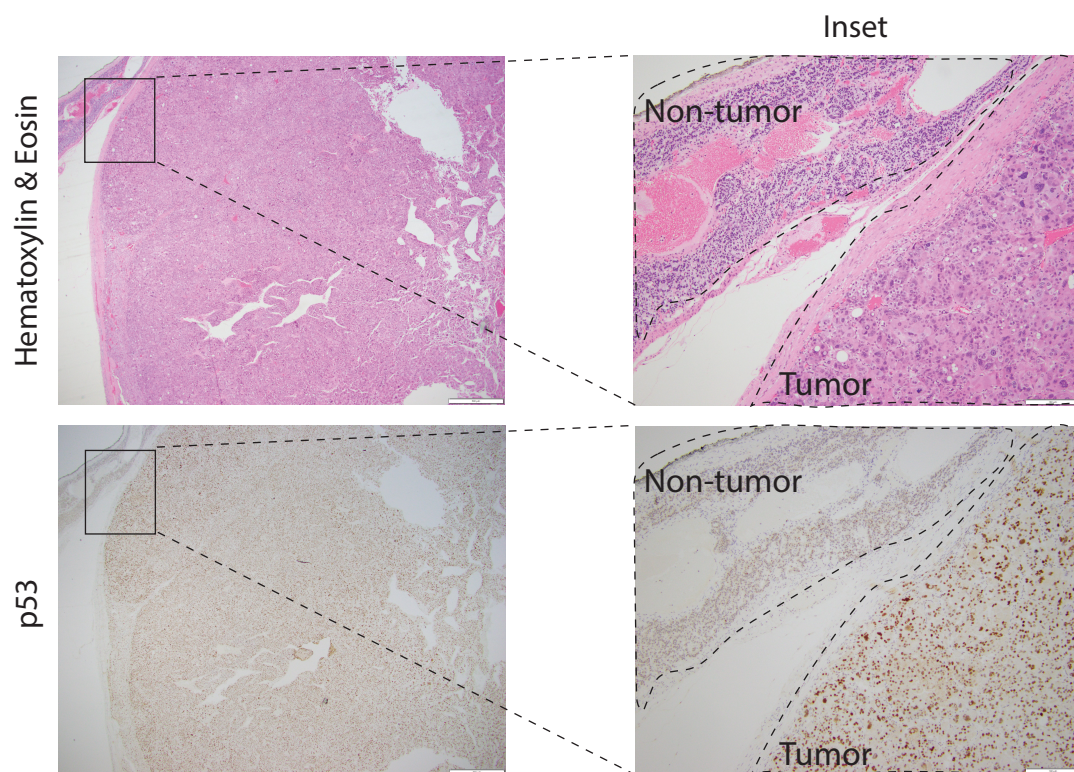

Supplement: S1 Fig — (Top) Immunohistochemical staining of the ACC showing that tumor cells were immunoreactive for synaptophysin and Melan-A, with increased Ki67; total tissue Ki67 positivity was estimated at 15%. (Bottom) Neoplastic tissue contained higher levels of total p53 protein than adjacent non-neoplastic tissue. (PDF) [file pone.0320036.s002.pdf]

A.

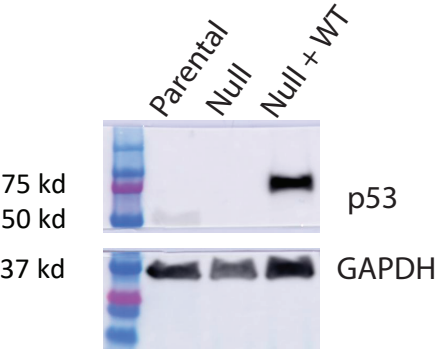

B.

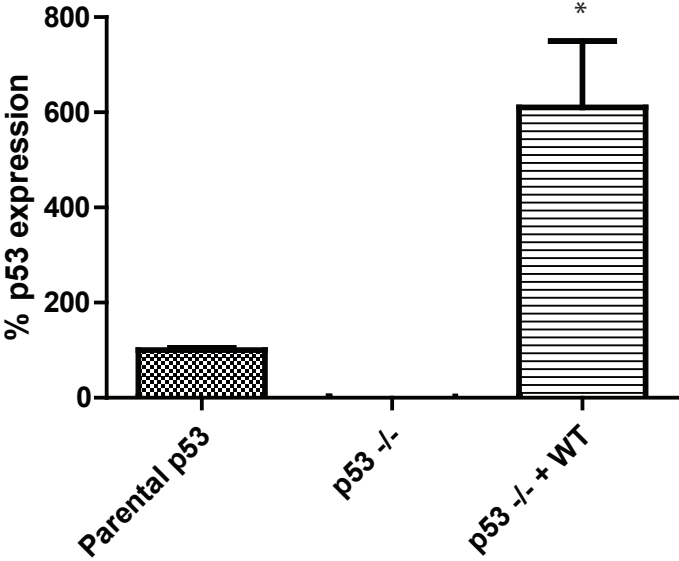

Supplement: S2 Fig — (A) A Western blot of HEK p53 +/+, HEK293 p53-/-, and WT-GFP-complemented HEK p53-/- cells, as well as a graphical depiction (B) of the relative abundances of p53 in each cell line. Data represent the mean expression levels, normalized to GAPDH, of at least three experimental replicates. (PDF) [file pone.0320036.s003.pdf]

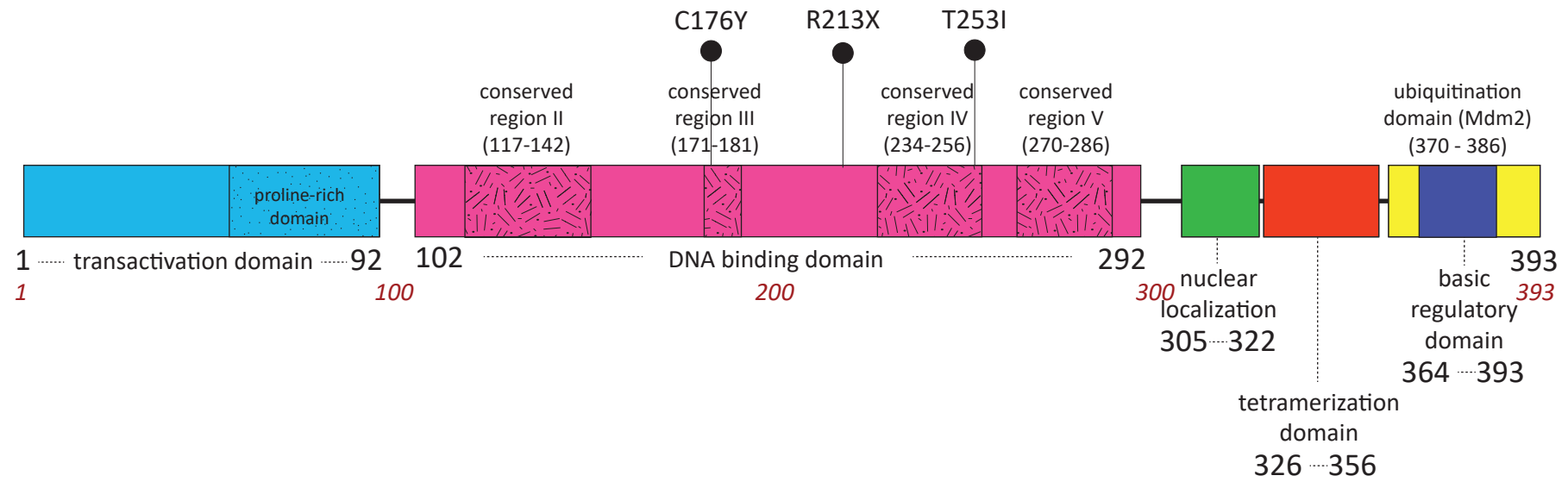

Supplement: S3 Fig — A cartoon schematic of the functional domains of the p53 protein overlaid with the locations of the mutations being examined in this study. Note that all three mutations lie within the DNA binding domain and that both single nucleotide polymorphisms (C176Y and T253I) are further located within highly conserved regions of the DNA binding domain of p53. (PDF) [file pone.0320036.s004.pdf]

**A.**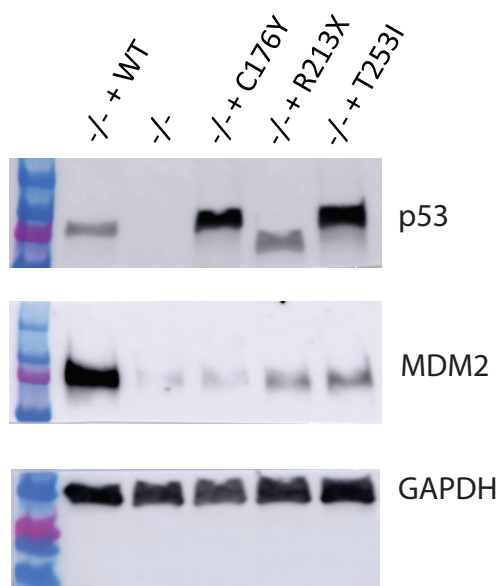**B.**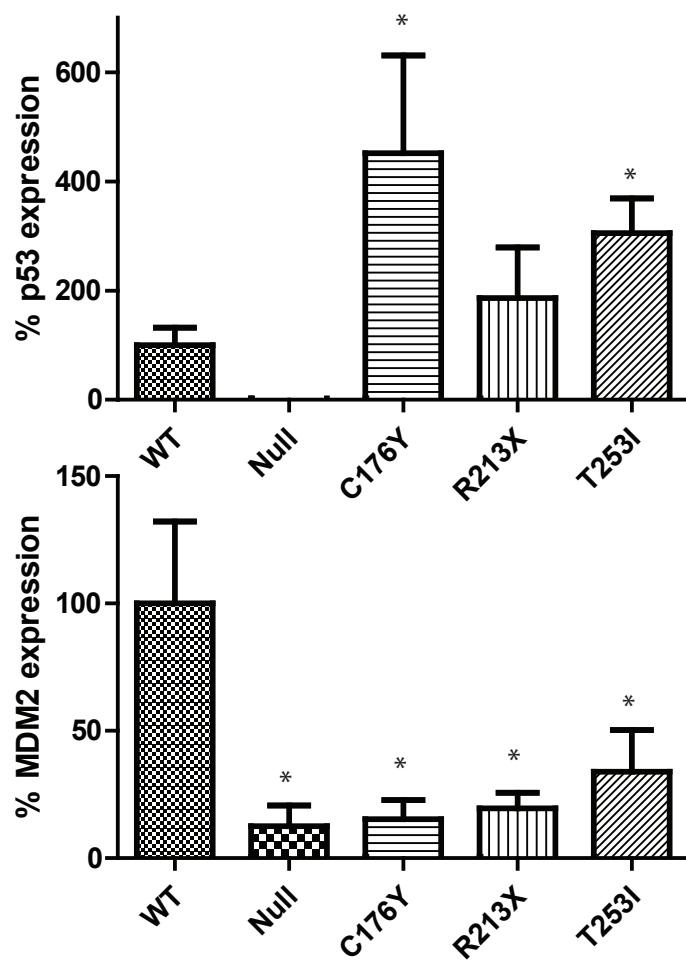

Supplement: S4 Fig — A Western blot of HEK p53-/- with or without complementation with WT-GFP, C176Y-GFP, R213X-GFP, or T253I-GFP p53 as well as a graphical depiction of the relative abundances (B) of p53 and MDM2 in each cell line. Data represent the mean expression levels, normalized to GAPDH, of three experimental replicates. (PDF) [file pone.0320036.s005.pdf]

A.

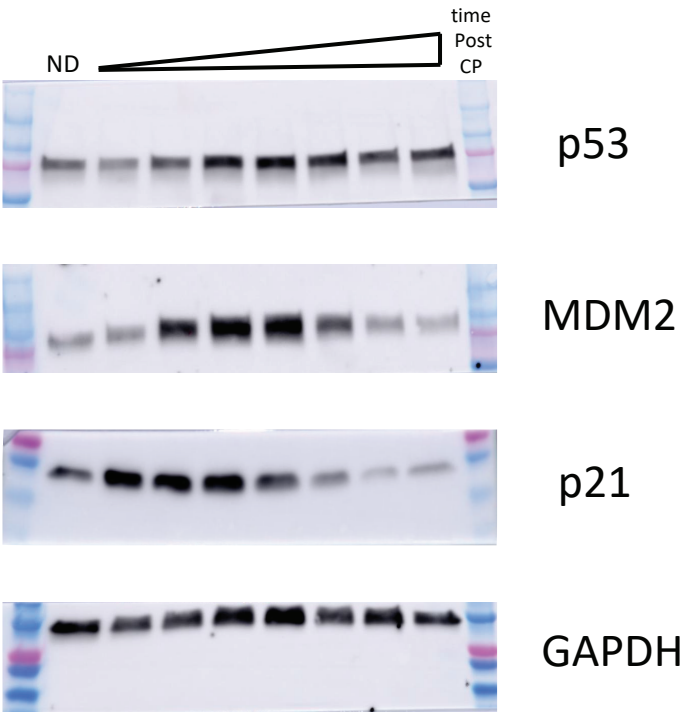

B.

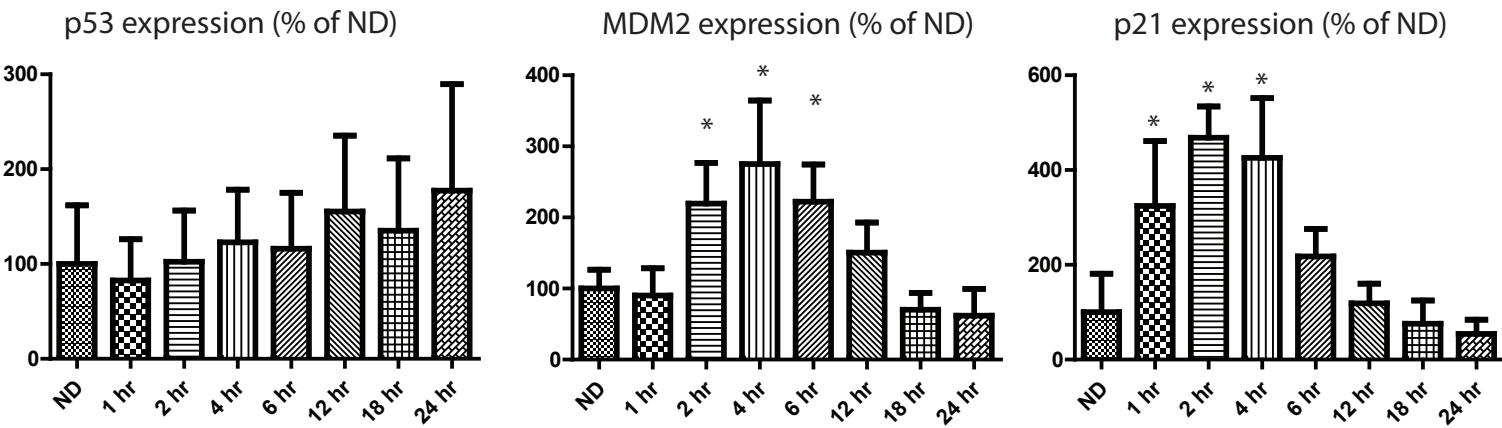

Supplement: S5 Fig — (A) WT-GFP-complemented HEK p53-/- cells were treated with 5 µg/mL for one hour (or untreated) and then allowed to recover for a period of time ranging from 1–24 hours. At the end of the recovery period, cells were harvested and a Western Blot was run to measure the expression of p53, MDM2, and p21, using GAPDH as a loading control. (B) A graphical depiction of the relative abundances of p53, MDM2, and p21, normalized to GAPDH, at each timepoint after damage for the duration of the recovery period. Data were presented as the percentage of expression relative to non-damaged (ND) cells and represent the average of three replicates. (PDF) [file pone.0320036.s006.pdf]

**A.**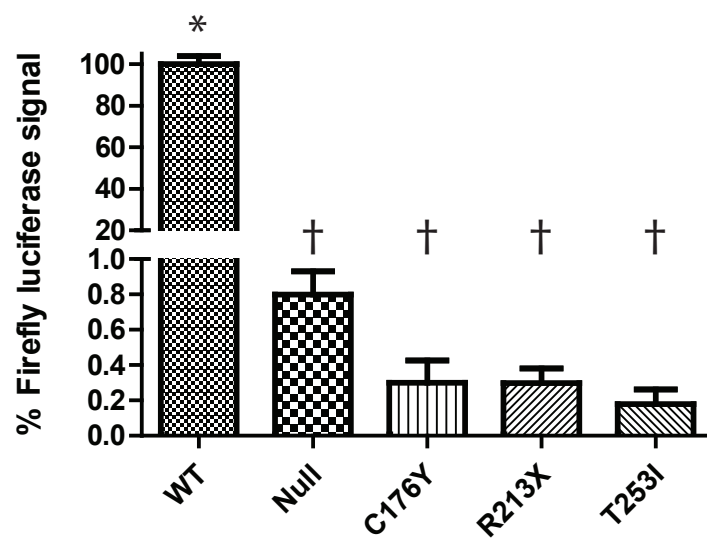**B.**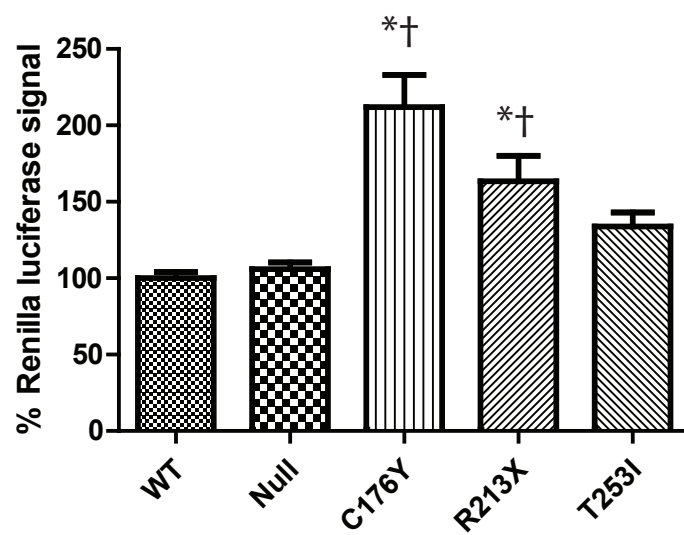

Supplement: S6 Fig — (A) p53-driven firefly luciferase reporter assay and (B) CMV-driven renilla reporter assay results that were combined to generate the ratio as seen in Fig 5b. Data represent the mean luciferase signal, normalized to p53 WT-GFP expressing cells, of three experimental replicates. Samples marked with an * are not significantly different from each other. (PDF) [file pone.0320036.s007.pdf]
