# Supplementary material for: Characterization of p53 p.T253I as a pathogenic mutation underlying Li-Fraumeni Syndrome
Source: PLoS One. 2025 Dec 5;20(12):e0320036. doi: 10.1371/journal.pone.0320036 (PMC12680149; doi:10.1371/journal.pone.0320036)
Supplement: Attachment — Submitted filename: Response to Reviewers for 2-25 PLOS One submission.docx [file pone.0320036.s008.docx]

PONE-D-25-07425

Characterization of p53 p.T253I as a pathogenic mutation underlying Li-Fraumeni Syndrome

**Response to Reviewers**

*1) We note that the current version of the manuscript includes tracked changes. Before we proceed with your submission, please upload a clean version after considering all the comments. Thank you for your attention.*

We have uploaded both a clean version as well as the tracked changes version since the original submission (PONE-D-24-56884, “Characterization of p53 p.T253I as a pathogenic mutation underlying Li-Fraumeni syndrome in an infant with adrenal cortical carcinoma”).

*2) Please provide a response to reviewers, including all the comments.*

The feedback we got from the original submission was “PLOS ONE has been specifically designed to communicate original research contributing to the base of academic knowledge. Individual case reports and collections of case reports are not considered for publication.”

Our initial submission included clinical data associated with the presentation of the patient whose tumor (and germline) TP53 status brought the T253I variant to our attention. This made it seem as though we had submitted a clinical case report, but this was not our intention. Rather, we are interested in determining the consequences of the T253I TP53 variant on canonical cellular p53-mediated damage signals and responses. Thus, we have removed the clinical aspects of the report in favor of focusing on the cellular and biochemical aspects of p53 signaling in cells, comparing wild type p53 with T253I p53 and including known loss-of-function variants as controls. We hope that by removing the clinical aspects of the case that the manuscript will be suitable for consideration in *PLOS One*.

We hope that the editors and reviewers will consider the changes in the revised document to be favorable. Thank you for re-considering our manuscript.

**John D'Orazio, M.D., Ph.D.**

Professor of Pediatrics and Chief, Division of Pediatric Hematology/Oncology

University of Kentucky College of Medicine

Combs Research Bldg. Rm. 204

800 Rose Street, Lexington, KY 40536-0096

Email: [jdorazio@uky.edu](mailto:jdorazio@uky.edu)

Office: (859) 218-0931; Fax: (859) 323-8641
