# Supplementary material for: Characterization of p53 p.T253I as a pathogenic mutation underlying Li-Fraumeni Syndrome
Source: PLoS One. 2025 Dec 5;20(12):e0320036. doi: 10.1371/journal.pone.0320036 (PMC12680149; doi:10.1371/journal.pone.0320036)
Supplement: Attachment — Submitted filename: Response to Reviewers for PLOS One resubmission - jd.docx [file pone.0320036.s009.docx]

**PONE-D-25-07425**

**Characterization of p53 p.T253I as a pathogenic mutation underlying Li-Fraumeni Syndrome**

**Response to Reviewers**

We sincerely thank the reviewers and editors for the opportunity to resubmit our manuscript entitled “Characterization of p53 p.T253I as a pathogenic mutation underlying Li-Fraumeni Syndrome” for publication in PLoS One. In this manuscript we identify the T253I mutation in p53 as a *bona fide* loss of function variant associated with adrenocortical carcinoma found in an infant suspected of having Li-Fraumeni syndrome. We found the comments of the reviewers useful and have made changes in our revised manuscript in accordance with their suggestions. We have attempted to answer point-by-point each of the reviewers’ specific concerns in the comments section below. We feel we have addressed all of the critiques and responded to the suggestions made by the reviewers and editors.

*1. Please ensure that your manuscript meets PLOS ONE's style requirements, including those for file naming.*

We have confirmed that our revised document conforms to PLOS ONE’s style requirements, including those for file naming, and we have adjusted our references list to be in the PLOS One style format.

*2. Thank you for stating the following financial disclosure: Kentucky Pediatric Cancer Research Trust Fund, NCI P30 CA177558, NIH P20 GM121327. Please state what role the funders took in the study. If the funders had no role, please state: "The funders had no role in study design, data collection and analysis, decision to publish, or preparation of the manuscript." If this statement is not correct you must amend it as needed.*

The funders had no role in study design, data collection and analysis, decision to publish, or preparation of the manuscript.

*Please include this amended Role of Funder statement in your cover letter; we will change the online submission form on your behalf.*

We have included an amended Role of Funder statement in our revised cover letter, and we thank the editors for changing the online submission form on our behalf.

*3. Thank you for stating the following in the Acknowledgments Section of your manuscript:*

*This research was supported by the Biostatistics and Bioinformatics, Flow Cytometry and Immune Monitoring, Biospecimen Procurement and Translational Pathology, Cancer Research and Informatics, and Oncogenomics Shared Resource Facilities of the University of Kentucky Markey Cancer Center, each supported by the University of Kentucky Markey Cancer Center’s Cancer Center Support Grant (P30 CA177558). Additional support was provided by the Kentucky Pediatric Cancer Research Trust Fund, the Joy Wills Endowment for Childhood Cancer Research, and the DanceBlue Philanthropic Organization. We thank the COBRE Imaging Core facility at the University of Kentucky supported by P20 GM121327 for providing service with microscopes and imaging systems. We are indebted to the Clinical Research Office of the Division of Pediatric Hematology/Oncology at the University of Kentucky for their efforts in accrual and data management for Project Inherited Cancer Risk, a clinical study to identify and manage children, adolescents and young adults with cancer predisposition syndromes.*

*We note that you have provided funding information that is not currently declared in your Funding Statement. However, funding information should not appear in the Acknowledgments section or other areas of your manuscript. We will only publish funding information present in the Funding Statement section of the online submission form. Please remove any funding-related text from the manuscript and let us know how you would like to update your Funding Statement. Currently, your Funding Statement reads as follows:*

*Kentucky Pediatric Cancer Research Trust Fund*

*NCI P30 CA177558*

*NIH P20 GM121327*

These funding sources are appropriately listed in the Funding Statement, and the shared resource (core) facilities noted in the Acknowledgments section each derive from either the NCI P30 or NIH P20 grants.

*Please include your amended statements within your cover letter; we will change the online submission form on your behalf.*

We have altered the Acknowledgement section to the following in the revised document, and we thank the editors for changing the online submission form on our behalf:

This research was supported by the Biostatistics and Bioinformatics, Flow Cytometry and Immune Monitoring, Biospecimen Procurement and Translational Pathology, Cancer Research and Informatics, and Oncogenomics Shared Resource Facilities of the University of Kentucky Markey Cancer Center. Additional support was provided by the Kentucky Pediatric Cancer Research Trust Fund, the Joy Wills Endowment for Childhood Cancer Research, and the DanceBlue Philanthropic Organization. We thank the COBRE Imaging Core facility at the University of Kentucky for providing service with microscopes and imaging systems. We are indebted to the Clinical Research Office of the Division of Pediatric Hematology/Oncology at the University of Kentucky for their efforts in accrual and data management for Project Inherited Cancer Risk, a clinical study to identify and manage children, adolescents and young adults with cancer predisposition syndromes. Finally, we are immensely grateful to the patients and families who consented to take part in the “Project Inherited Cancer Risk” study at the University of Kentucky by which these variants were identified.

*4. When completing the data availability statement of the submission form, you indicated that you will make your data available on acceptance. We strongly recommend all authors decide on a data sharing plan before acceptance, as the process can be lengthy and hold up publication timelines. Please note that, though access restrictions are acceptable now, your entire data will need to be made freely accessible if your manuscript is accepted for publication. This policy applies to all data except where public deposition would breach compliance with the protocol approved by your research ethics board. If you are unable to adhere to our open data policy, please kindly revise your statement to explain your reasoning and we will seek the editor's input on an exemption. Please be assured that, once you have provided your new statement, the assessment of your exemption will not hold up the peer review process.*

We agree to data sharing, and have indicated that in the resubmission process*.*

*5. Please review your reference list to ensure that it is complete and correct. If you have cited papers that have been retracted, please include the rationale for doing so in the manuscript text, or remove these references and replace them with relevant current references. Any changes to the reference list should be mentioned in the rebuttal letter that accompanies your revised manuscript. If you need to cite a retracted article, indicate the article’s retracted status in the References list and also include a citation and full reference for the retraction notice.*

We have reviewed the reference list for accuracy; none have been retracted.

*Responses to Reviewer's Questions*

*Reviewer #1:*

*1. The design of this study does not exactly replicate the genetics of LFS, in which the mutant TP53 allele is heterozygous with a wild-type allele. This raises the question as to whether any of the 3 mutant alleles studied here may behave differently in these assays if their wild-type partner was present?*

We agree that our study design does not investigate the impact of each variant studied on cellular damage and survival responses in the heterozygous setting which would model the germline LFS state. For this paper, we decided to interrogate the T253I variant to understand its impact on p53 responses in isolation, but are planning follow-up studies (for a future manuscript) to determine how T253I p53 interacts with wt p53 and its impact on cell responses in a heterozygous setting. We have included this statement in the discussion in the revised manuscript. Additionally, It is worth noting that the ACC that contained the T253I mutation developed a loss of heterozygosity with a mutant allelic frequency of over 75%, consistent with other observations that p53 pathogenicity often requires the loss of the remaining wt allele. Therefore, studying the mutant in a homozygous setting may more accurately reflect the tumor cell physiology

*2. Since the 2 known pathogenic TP53 alleles are such a critical component of this analysis, it would be preferable for them to appear in the Introduction, along with appropriate referencing. The reference provided where they're currently introduced in the Discussion (Zhang et al., 2014) appears to only support the pathogenicity of R213X, not C176Y.*

We thank the reviewer for this suggestion, and have modified the manuscript accordingly, including providing referencing to each of the known pathogenic TP53 alleles.

*3. There is a major statistical issue for most of the bar charts, apparently with designation of significance. It appears that whatever symbols supposed to appear were substituted by undefined a-d lettering. In addition, once these designations are corrected, the p-values they're associated with and what bars they reference needs to be defined in every Figure Legend where they're shown.*

We apologize for this, and have modified the figures accordingly. Specifically, we have changed the alphabetic system to a system in which significant departure from WT or null condition is indicated with a symbol and have indicated p-values on the graphs or in the figure legends.

*4. In Fig. 1B, the Null & WT bars should be swapped to match order of all other Figs.*

We thank the reviewer for this insight and have now modified Fig. 1B to match the order of the other figures.

*5. In Fig. S-2B, the 'Parental p53' designation is confusing & vague. Perhaps just 'WT p53' or 'p53 +/+' might serve better?*

We agree, and have replaced “parental p53” with “p53 ^+/+^”.

*Reviewer #2: 1. It would be important to show whether there is any difference in function between GFP-tagged vs untagged P53?*

We realize that tagging p53 proteins with GFP may alter their behavior, however we observed that GFP-tagged p53 WT cells complement the loss of p53 signaling seen in p53 -/- cells, and GFP-tagged p53 mutant cells exhibit attenuated responses. Since each p53 mutant contains the identical GFP tag as the wt p53, it is unlikely that the presence of the GFP tag accounts for attenuated responses of the p53 mutants. While we fully appreciate that p53 responses seen in cells harboring GFP-tagged complementation variants may differ from with untagged variants, we feel this issue does not detract from the primary observation in the manuscript (i.e. that T253I p53 exhibits loss of function). We have added this point to the Discussion section.

*2. Although authors stated that p53 T253I has less transcriptional activity due to inefficient DNA binding through an ELISA-based assay, it would be more convincing to show with ChIP-qPCR for a couple of known p53-dependent regulatory genes whether such observation still holds for a complementary assay system.*

We have performed ChIP-qPCR assays using the p53 binding site of the p21 gene and included it in our revised manuscript (please see Figure 5b). Importantly, the ChIP data correlate with the p53 binding ELISA data.

*6. PLOS authors have the option to publish the peer review history of their article (what does this mean?). If published, this will include your full peer review and any attached files.*

We have no objections to publishing the peer review history of the article.

*While revising your submission, please upload your figure files to the Preflight Analysis and Conversion Engine (PACE) digital diagnostic tool, https://pacev2.apexcovantage.com/. PACE helps ensure that figures meet PLOS requirements. To use PACE, you must first register as a user. Registration is free. Then, login and navigate to the UPLOAD tab, where you will find detailed instructions on how to use the tool. If you encounter any issues or have any questions when using PACE, please email PLOS at figures@plos.org. Please note that Supporting Information files do not need this step.*

Figures have been run through PACE and formatted accordingly.

We hope that the editors and reviewers will consider the changes in the revised document to be favorable. Thank you for your time and expertise in evaluating our work and for re-considering our manuscript.

**John D'Orazio, M.D., Ph.D.**

Professor of Pediatrics and Chief, Division of Pediatric Hematology/Oncology

University of Kentucky College of Medicine

Combs Research Bldg. Rm. 204

800 Rose Street, Lexington, KY 40536-0096

Email: [jdorazio@uky.edu](mailto:jdorazio@uky.edu)

Office: (859) 218-0931; Fax: (859) 323-8641
